# War Office Increases Payments to Hospitals

**Published:** 1918-02-09

**Authors:** 


### WAR OFFICE INCREASES PAYMENTS TO HOSPITALS.

Most hospital managers will recognise the difficulties which have been successfully overcome by the negotiations between the War Office and the British Hospitals Association. The result secures an increase in the capitation allowance paid to voluntary hospitals for sick and wounded sailors and soldiers at the rate of 9d. per occupied bed per day, with 6d. per unoccupied bed per day, both dating as from October 1, 1917. On the question of a differential rate for certain hospitals an agreement has been come to that it will be best to lay down a flat rate for all hospitals. Further, if any hospital can show special circumstances to distinguish it from other hospitals, such hospital is directed to make a special application to the War Office, not for any increase in the flat rate, but for a special donation. In order to clear the way for those hospitals which decide to make such special applications, the British Hospitals Association has in preparation full details of the arrangements made, in which details of the special circumstances will be instanced, as, for example, those for a fully equipped pathological department, special massage facilities, and similar particulars relating to the grounds on which the special application is based.

We believe experience will prove that the agreement come to between the War Office, and the British Hospitals Association representing the voluntary hospitals, includes a wide enough basis to facilitate a satisfactory arrangement to meet all cases, and to secure full justice to the parties represented in the agreement arrived at. It is of the first importance that full justice should be done all round. The hospitals will welcome the results secured through the instrumentality of the British Hospitals Association, and will recognise the value which attaches to its active intervention. Further, it will be well to remember that the great changes which are likely to take place in the arrangements and in the extension of the area of usefulness of each voluntary hospital, due to the altered conditions arising after the war, warrant early steps being taken to place the management of the British Hospitals Association on the widest possible basis, so that its representative character and strength, as an administrative centre, representative of the best hospital opinion, may be secured and cemented.

Reverting to the treatment of disabled sailors and soldiers, we have this week some important additional information as to the methods of procedure, with which all voluntary hospital managers should hasten to make themselves familiar. The Ministry of Pensions has issued a revised list (No. 2) of Local War Pensions Committees, 1917, which contains an alphabetical list of local committees for county boroughs and urban districts, showing the county in which they are situated, throughout England and Wales, Scotland, and Ireland. It also includes a list of local committees arranged alphabetically in county order. Every hospital should obtain a copy of List No. 2 without delay. The honorary secretaries of the British Hospitals Association, see page 399, request that the voluntary hospitals in London, which are willing to undertake the treatment of disabled sailors and soldiers, will communicate direct with the County Local Committee, whose address is 43 Bloomsbury Square, W.C. 1. It will be well to bear in mind in this connection that there are forty-five sub-committees in London, but the central body, the County Local Committee, alone has power to conclude valid arrangements. The hospitals should clearly understand this point, as it cannot fail to save them unnecessary trouble and correspondence. The honorary secretaries further make it plain that the meetings and resolutions of the governors and staffs of the large London hospitals, who had considered this question, were respectively held and passed quite independently of the meetings and resolutions held and passed by the Council of the British Hospitals Association. Still, the latter body properly feel it would be less than justice not to acknowledge the assistance which the resolutions of these large London hospitals have been to the Council, in their careful endeavours to arrive at some common principles of action, in dealing with the Ministry of Pensions.

We share the view that the best treatment that the country can provide for disabled sailors and soldiers is to be found in, or in connection with, the voluntary hospitals, a fact which the governors and staffs of these institutions fully realise and welcome, on account of the opportunities it offers for good and inspiring responsibilities. The more the difficulties which presented themselves are realised, the greater is likely to be the feeling of appreciation to the representatives of the British Hospitals Association and the London hospitals, who jointly with the representatives of the War Office and of the Ministry of Pensions, have succeeded in securing a provisional settlement to last for one year. This first step is full of promise. We see in it the forecast of permanent arrangements that will not only work smoothly but fulfil in practice the country's wish, that they shall in fact prove to be the expression of a high and appealing principle, and prove the fulfilment of the nation's grateful and pious duty towards its disabled sailors and soldiers.

